# Oxidative Stress Promotes Instability of Regulatory T Cells in Antineutrophil Cytoplasmic Antibody-Associated Vasculitis

**DOI:** 10.3389/fimmu.2021.789740

**Published:** 2021-12-07

**Authors:** Yasuhiro Shimojima, Dai Kishida, Takanori Ichikawa, Ryota Takamatsu, Shun Nomura, Yoshiki Sekijima

**Affiliations:** Department of Medicine (Neurology and Rheumatology), Shinshu University School of Medicine, Matsumoto, Japan

**Keywords:** regulatory T cells, reactive oxygen species, FoxP3, ANCA-associated vasculitis, effector cytokines, plasticity, mTOR, resveratrol

## Abstract

We investigated the characteristics of regulatory T cells (Tregs), focusing on the relationship between their stability and reactive oxygen species (ROS), in antineutrophil cytoplasmic antibody-associated vasculitis (AAV). Intracellular expressions of effector cytokines, forkhead box protein 3 (FoxP3), ROS, phosphorylated mammalian target of rapamycin (mTOR), and sirtuin 1 (SIRT1) in Tregs from peripheral blood mononuclear cells (PBMCs) of patients with AAV and healthy controls (HC) were analyzed. The alterations in and functional ability of Tregs were compared before and after resveratrol (RVL) treatment of PBMCs in patients with AAV. Significantly higher expressions of interferon (IFN)-γ, interleukin (IL)-17, IL-4, ROS, and phosphorylated mTOR (pho-mTOR) and lower expression of SIRT1 in CD4+CD25+FoxP3+ cells were found in patients with AAV than in the HC. FoxP3 expression in CD4+CD25+ cells and suppressive function of Tregs were significantly lower in patients with AAV than in the HC. Tregs after RVL treatment demonstrated significant decreases in IFN-γ, ROS, and pho-mTOR levels and increases in FoxP3, SIRT1 levels, and functional activity. Conversely, the direct activation of SIRT1 by SRT1720 resulted in decreased FoxP3 expression, with no reduction in ROS levels. The pho-mTOR levels were significantly higher in Tregs after activation by SRT1720 than in those after RVL treatment. This study suggested that imbalanced changes in Tregs could be attributed to mTOR activation, in which ROS overproduction was predominantly implicated. Therefore, ROS is a key mediator for promoting Tregs instability in AAV.

## Introduction

Antineutrophil cytoplasmic antibody (ANCA)-associated vasculitis (AAV) is a systemic autoimmune disorder involving a pauci-necrotizing small-sized vasculitis, in which ANCA targeting myeloperoxidase (MPO) and proteinase 3 (PR3) participate in the pathogenesis of the disease. Suggestive etiologies, including various environmental factors and multifactorial susceptibility genes, have been shown to date (1). Moreover, divergent pathological mechanisms in both innate and acquired immune systems are implicated in the development of AAV, suggesting that inflammatory damage of targeted organs involving vasculitis and granuloma formation is promoted by hyperactivated immunocompetent cells, such as neutrophils, macrophages, autoreactive B cells, and T cells, as well as proinflammatory cytokines and reactive oxygen species (ROS) (1, 2). Some studies have also reported the dysfunction and imbalance of regulatory T cells (Tregs) in AAV (3–5). Additionally, the plasticity of Tregs involving a shift to helper T (Th)-like cells by intracellular expression of effector cytokines is induced in inflammatory autoimmune diseases (6, 7). However, it remains uncertain how the plastic changes of Tregs are evoked in AAV, although it has been hypothesized that dysfunction of Tregs might be exerted by exposure to effector cytokines, especially conversion to the Th17-lineage (8). Hyperexpression of Th cells is strongly implicated in the development of AAV, suggesting that the etiologic factors related to AAV could lead to the abrogation of the intracellular signaling of Tregs, such as forkhead box P3 (FoxP3) expression. Oxidative stress adversely affects the expression and functional ability of Tregs in the pathogenic mechanisms of systemic lupus erythematosus (SLE) (9). The plasticity of T cells is physiologically promoted under enhanced oxidative stress; moreover, the expression of effector cytokines may be altered depending on the concentration of ROS (10, 11). Given these immune reactions and the pathogenesis of AAV, it is necessary to consider that oxidative stress may be a mediator affecting the conditions of Tregs because oxidative stress plays a crucial role in the development of AAV (2). The mammalian target of rapamycin (mTOR) pathway can be affected by metabolic alterations, including oxidative stress (12). In the development of Tregs, mTOR activation inhibits FoxP3 expression, which is also associated with the induced expression of effector cytokines in T cells (12). Accordingly, it is necessary to investigate the intracellular circumstances of ROS expression and mTOR activation as clues for promoting the plasticity of Tregs in AAV. It is still uncertain how oxidative stress affects the kinetics of Tregs in AAV.

Herein, we investigated the characteristics of Tregs, focusing on their imbalanced alteration, including expression of effector cytokines, ROS, mTOR activation, and their functional ability. Additionally, we evaluated the stability of Tregs after treatment with resveratrol (RVL), a phenolic compound that can potentially exert antioxidant, anti-immune aging, and anti-inflammatory effects (13, 14). RVL is also known as a potential activator of sirtuin (silent mating type information regulating 2 homolog) 1 (SIRT1), which is a nicotinamide adenosine dinucleotide (NAD)^+^-dependent histone/protein deacetylase that serves as a substrate for stabilizing mammalian physical functions (15). SIRT1 also regulates inflammatory and metabolic reactions of immunocompetent cells as an anti-immune aging and homeostasis mediator (16, 17). RVL may be an anti-aging therapy. Therefore, we evaluated SIRT1 expression in Tregs. To the best of our knowledge, this is the first attempt to investigate the characteristics of circulating Tregs, focusing on their plasticity and oxidative damage in AAV.

## Material and Methods

### Patients

Twenty-five patients with microscopic polyangiitis (MPA) or granulomatosis with polyangiitis (GPA) who had not received immunosuppressive therapy were enrolled in this study. The diagnosis and classification of MPA or GPA were determined according to the criteria of the Chapel Hill Consensus Conference (18) and/or the consensus algorithm proposed by the European Medicines Agency (19). Patients with complications of neoplasms or infections were excluded from the study. Of the 25 patients (mean age, 63 years; 8 men and 17 women), 14 (56%) and 11 (44%) were classified as MPA and GPA, respectively. The Birmingham Vasculitis Activity Score (BVAS) (20) was 19.2 ± 6.7. The related symptoms based on BVAS and laboratory findings, which included white blood cell count, serum levels of C-reactive protein, erythrocyte sedimentation rate, and presence of MPO-ANCA or PR3-ANCA, were also evaluated before initiating treatment (**Supplementary Table 2**). For comparison, 17 age-matched healthy controls (HC), with a mean age of 58 years (seven men, 10 women), were included in the control group. Whole blood samples were obtained from 25 patients prior to initiating immunosuppressive therapy and 17 HC enrolled in this study. The local Ethics Committee of Shinshu University approved this study (approval number: 614). All participants provided written informed consent.

### Cell Isolation and Quantitative Real-Time Polymerase Chain Reaction

Peripheral blood mononuclear cells (PBMCs) were isolated from whole blood samples collected in EDTA-coated tubes by gradient centrifugation with Ficoll-Hypaque PLUS (GE Healthcare, Pittsburgh, PA, USA). The CD4+CD25+ regulatory T cell isolation kit (Miltenyi Biotec, Bergisch Gladbach, Germany) isolated Tregs from unstimulated PBMCs. Total RNA was extracted from isolated Tregs using an RNeasy Mini kit (Qiagen, Venlo, Netherlands). Complementary DNA (cDNA) was synthesized using the Maxima First-Strand cDNA Synthesis Kit (Thermo Scientific, Waltham, MA, USA). cDNA was used to perform qRT-PCR with the StepOnePlus Real-Time PCR System (Applied Biosystems, Foster City, CA, USA) using SYBR Premix Ex Taq II (Takara, Kusatsu, Japan). The primers used were as follows: glyceraldehyde-3-phosphate dehydrogenase (GAPDH) (QT00079247 [Hs_GAPDH_1_SG]), FoxP3 (QT00048286 [Hs_FoxP3_1_SG]) (both from Qiagen), and mTOR (qHsaCID0012480 [ENSG00000198793]) (Bio-Rad, Hercules, CA, USA). In order to evaluate the results in qRT-PCR quantitatively, relative copy number (RCN) was calculated using threshold cycle (Ct) of GAPDH and target gene as follows: ΔCt = Ct (target gene) – Ct (GAPDH); RCN = 2^-ΔCt^.

### Cell Treatment and Flow Cytometry

Isolated PBMCs were incubated on a 24-well plate (1×10^6^/well) with and without 100 μM RVL (Sigma-Aldrich, St. Louis, MO, USA) or 5 μM SRT1720 (Abcam, Cambridge, UK) at 37 ° for 24 h. Incubated PBMCs were stimulated with 0.5 μg/ml ionomycin, 0.04 μg/ml phorbol myristate acetate (both from Sigma-Aldrich), and 2 μM monensin (BD Biosciences, San Diego, CA, USA) at 37°C for 4 h. Stimulated PBMCs were stained with PE/Cy7-conjugated anti-CD4 (BioLegend, San Diego, CA, USA) and PC5-conjugated anti-CD25 (Beckman Coulter, Brea, CA, USA) with or without PE-conjugated anti-CD152 (cytotoxic T-lymphocyte-associated protein 4, CTLA-4) (Beckman Coulter) antibodies. The stained PBMCs were permeabilized with Cytofix/Cytoperm (BD Biosciences) and then stained with FITC-conjugated anti-IFN-γ (Beckman Coulter), PE-conjugated anti-IL-17 (BD Biosciences), PE-conjugated anti-IL-4 (Beckman Coulter), FITC-conjugated transforming growth factor (TGF)-β1 (BioLegend), PE-conjugated IL-10 (BioLegend) antibodies, or PE-conjugated mTOR (pS2448) antibody (BD Biosciences) for detecting phosphorylated mTOR (pho-mTOR), as well as PE-conjugated (BD Biosciences), FITC-conjugated, or Pacific blue-conjugated anti-FoxP3 (both from BioLegend). Alternatively, permeabilized PBMCs were stained with Alexa Fluor 405-conjugated anti-SIRT1 (Novus Biologicals, Littleton, CO, USA) and PE-conjugated anti-FoxP3. Treated cells were acquired on a FACSCanto II flow cytometer (BD Bioscience), and the data were analyzed using FlowJo software version 7.6.5 (Tree Star Inc., Ashland, OR, USA).

### Measurement of Intracellular ROS

PBMCs incubated with and without RVL or SRT1720 were stimulated with 200 μM tert-butyl hydroperoxide at 37 °C for 60 min. Treated PBMCs were fixed and permeabilized using FoxP3-staining buffer set (BD Bioscience) after staining with CellROX Deep Red Reagent (Invitrogen, Carlsbad, CA, USA), PE/Cy7-conjugated anti-CD4, and PC5-conjugated anti-CD25 antibodies. Permeabilized PBMCs were stained with PE-conjugated FoxP3. Intracellular ROS was detected on a flow cytometer and analyzed using FlowJo software.

### Suppression Assay of Tregs

Suppression assays were performed to evaluate the suppressive ability of Tregs. Tregs were isolated from incubated PBMCs with or without RVL using the CD4+CD25+ regulatory T cell isolation kit. To detect the target cells for Tregs, conventional T (Con-T) cells (CD4+CD25- cells) were isolated from untreated PBMCs of HC. Allogenic Con-T cells labeled with carboxyfluorescein succinimidyl ester (CFSE; 2μM, Invitrogen) and Tregs were co-cultured with anti-CD3/CD28 microbeads (Invitrogen) at a ratio of 1:1:1 in a 96-well U-bottom plate at 37°C for 4 d. The proliferation of Con-T cells was determined by CSFE dilution, and was acquired on a flow cytometer. The data were analyzed using FlowJo software.

### Statistical Analysis

All data are presented as the mean ± standard deviation (SD). The Mann-Whitney U test and Fisher’s exact probability test were used to compare the two independent groups. Consecutive data with and without treatment were compared using the Wilcoxon signed-rank test. The Kruskal-Wallis test was performed for comparisons among three independent groups, and the Steel-Dwass test was used for multiple comparisons. Statistical significance was defined as a *p*-*value* less than 0.05. All statistical analyses were performed using BellCurve for Excel (SSRI, Tokyo, Japan).

## Results

### Frequency of Tregs and Their Intracellular Expression of Effector Cytokines in AAV

**Figure 1:**
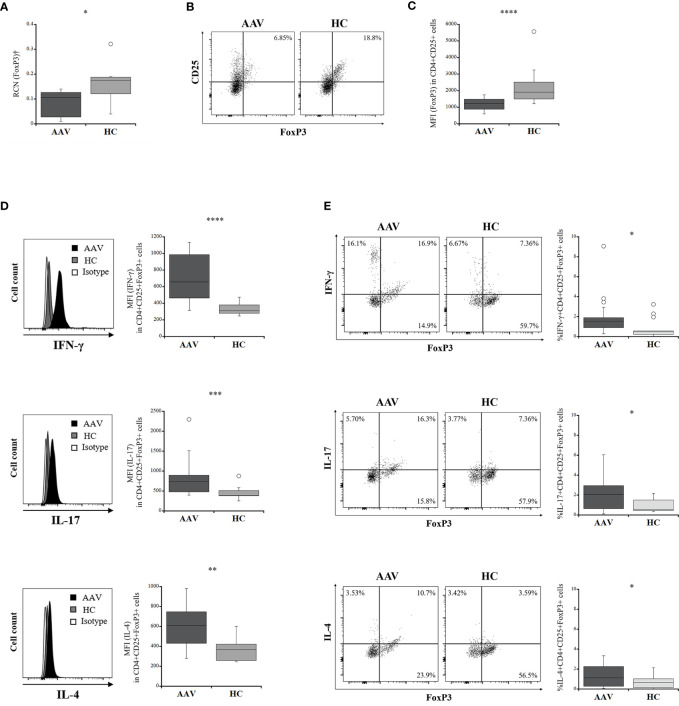
Comparisons of intracellular expression of FoxP3 and effector cytokine levels in regulatory T cells (Tregs) between the patients with ANCA-associated vasculitis (AAV) and healthy controls (HC). **(A)** relative copy number (RCN) of FoxP3 in isolated Tregs (AAV, n = 10; HC, n = 7). **(B)** representative dot plots showing CD25+FoxP3+ in CD4+ cells. **(C)** the median fluorescence index (MFI) of FoxP3 in CD4+CD25+ cells (AAV, n = 18; HC, n = 15). **(D)** representative histograms showing expression of interferon-γ (IFN-γ), interleukin (IL)-17, or IL-4 in CD4+CD25+FoxP3+ cells (left). MFI of IFN-γ, IL-17, or IL-4 in CD4+CD25+FoxP3+ cells (AAV, n = 18; HC, n = 15) (right). **(E)** representative dot plots showing expression of FoxP3 and interferon-γ (IFN-γ), interleukin (IL)-17, or IL-4 in CD4+CD25+ cells (left). Percent frequencies of IFN-γ, IL-17, or IL-4 positive CD4+CD25+FoxP3+ cells (AAV, n = 18; HC, n = 15) (right). †Isolated Tregs from peripheral blood mononuclear cells. *p < 0.05; **p < 0.005; ***p < 0.0005; ****p < 0.0001.

The percentage frequency of circulating Tregs (CD4+CD25+FoxP3+ cells) was significantly lower in the patients with AAV than in the HC (*p* = 0.0004) (**Table 1**). FoxP3 expression in CD4+CD25+ population and relative copy number (RCN) of FoxP3 in isolated Tregs were significantly lower in the patients with AAV than in the HC (*p <*0.0001, *p* = 0.040, respectively) (**Figures 1A‒C**). Intracellular expression of IFN-γ, IL-17, and IL-4 in Tregs was significantly higher in the patients with AAV than that in the HC (median fluorescence index [MFI]: *p* < 0.0001, *p* = 0.0003, *p* = 0.0009, respectively) (**Figure 1D**) (frequency: *p* = 0.002, *p* = 0.032, *p* = 0.004, respectively) (**Table 2**). The percent frequencies of IFN-γ, IL-17, and IL-4 positive CD4+CD25+FoxP3+ cells were significantly higher in the patients with AAV than in the HC (*p* = 0.009, *p* = 0.039, *p* = 0.008, respectively) (**Figure 1E**). In the additional analyses of CD4+CD25+CD127-/lowCD45RA+FoxP3+ cells, the percent frequency of them was significant lower in the patients with AAV than the HC (*p* = 0.025) (**Supplementary Table 3**). FoxP3 expression in CD4+CD25+CD127-/lowCD45RA+ cells was also significantly lower in the patients with AAV than in the HC (*p* = 0.0001), and expression of effector cytokines, including IFN-γ, IL-17, and IL-4, in CD4+CD25+CD127-/lowCD45RA+FoxP3+ cells were significantly higher in patients with AAV than in the HC (MFI: *p* < 0.0001, *p* = 0.003, *p* < 0.0001, respectively) (**Supplementary Figure 1**) (frequency: *p* < 0.0001) (**Supplementary Table 3**). Meanwhile, in comparison of intracellular expression of IFN-γ, IL-17, and IL-4 in high-and low-density expression of FoxP3 in the patients with AAV, their expression were significantly higher in the population of FoxP3high than in that of FoxP3low (*p* = 0.0001) (**Supplementary Figure 2**). In the HC, intracellular expression of IFN-γ, IL-17, and IL-4 was not significantly different in two distinct population of FoxP3 (*p* = 0.280, *p* = 0.306, *p* = 0.864, respectively).

**Table 1:** Frequency of CD4+CD25+FoxP3+ cells in patients with AAV and healthy controls.

		AAV	HC	*p-*value
		(n = 25)	(n = 17)	
In total lymphocytes				
	% CD4+ cells	48.99 ± 18.09	47.41 ± 9.09	0.682
	% CD4+CD25+ cells	21.19 ± 13.35	21.46 ± 11.57	0.729
	% CD4+CD25+FoxP3+ cells	5.34 ± 3.83	10.68 ± 6.76	0.0004

AAV, ANCA-associated vasculitis; HC, healthy controls. Data are presented as the mean ± SD.

**Table 2:** Frequencies of intracellular cytokines in Tregs in patients with AAV and healthy controls.

		AAV	HC	*p* value
		(n = 18)	(n = 15)	
In CD4+CD25+FoxP3+ cells				
	%IFN-γ	31.09 ± 23.34	8.38 ± 10.41	0.002
	%IL-17	35.05 ± 30.92	9.85 ± 14.33	0.032
	%IL-4	21.54 ± 21.83	2.47 ± 2.44	0.004

Tregs, regulatory T cells; AAV, antineutrophil cytoplasmic antibody-associated vasculitis; HC, healthy controls; IFN-γ, interferon-γ; IL, interleukin.

Data are presented as mean ± SD.

Accordingly, these results suggested that significant increases in intracellular expression of effector cytokines and decreased FoxP3 expression are characteristics of Tregs in patients with AAV.

### Intracellular Mediators Affecting Plasticity of Tregs in AAV

**Figure 2:**
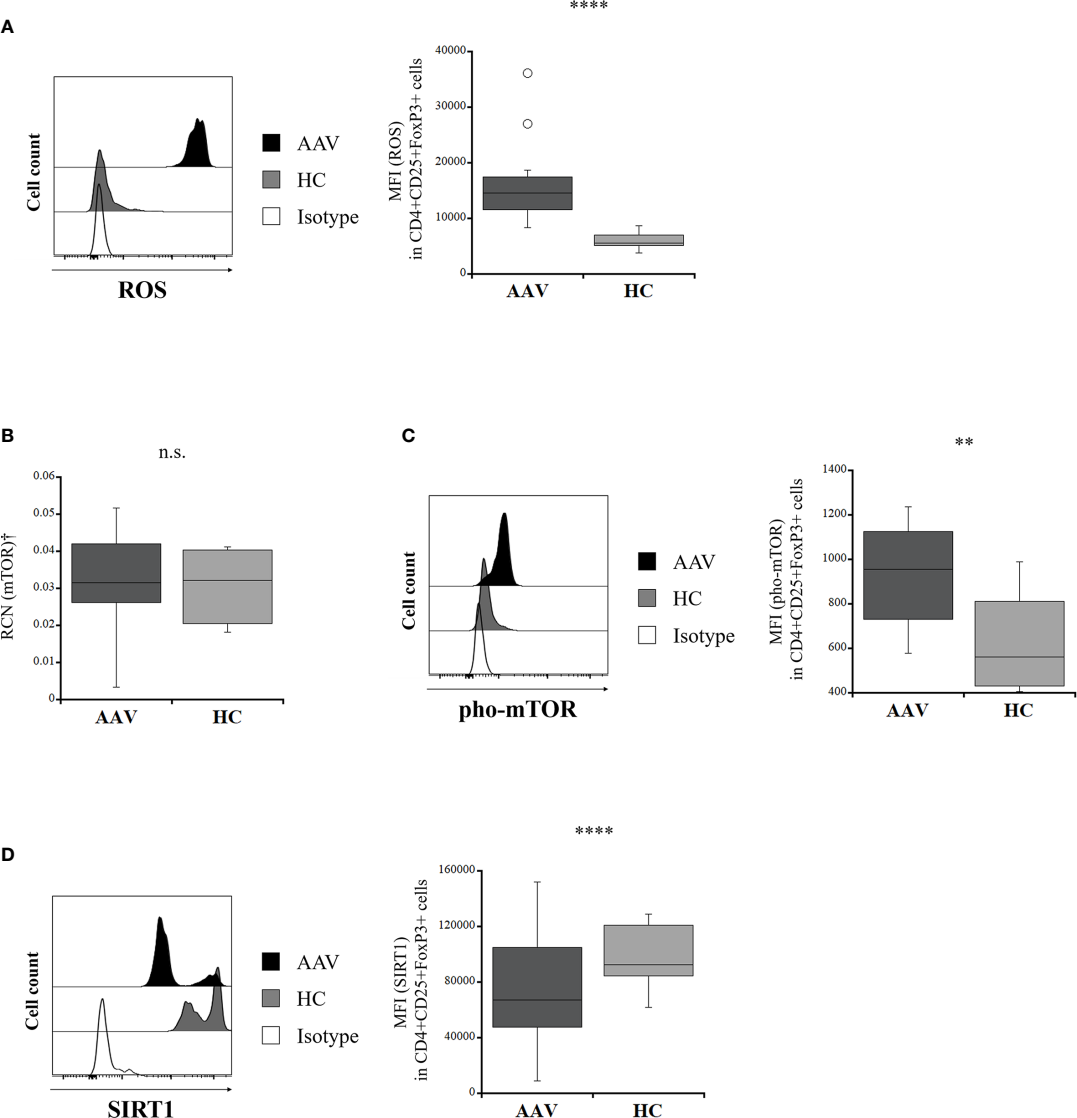
Comparison of intracellular mediators in regulatory T cells between the patients with ANCA-associated vasculitis (AAV) and healthy controls (HC). **(A)** representative histogram showing expression of reactive oxygen species (ROS) in CD4+CD25+FoxP3+ cells (left). The median fluorescence index (MFI) of ROS in CD4+CD25+FoxP3+ cells (AAV, n = 20; HC, n = 13) (right). **(B)** relative copy number (RCN) of mammalian target of rapamycin (mTOR) in isolated Tregs (AAV, n = 10; HC, n = 7). **(C)** representative histogram showing expression of phosphorylated mTOR (pho-mTOR) in CD4+CD25+FoxP3+ cells (left). MFI of pho-mTOR in CD4+CD25+FoxP3+ cells (AAV, n = 18; HC, n = 12) (right). **(D)** representative histogram showing expression of sirtuin 1 (SIRT1) in CD4+CD25+FoxP3+ cells (left). MFI of SIRT1 in CD4+CD25+FoxP3+ cells (AAV, n = 23; HC, n = 14) (right). †Isolated Tregs from peripheral blood mononuclear cells. n.s., not significant; **p < 0.005; ****p < 0.0001.

ROS production in Tregs was significantly greater in the patients with AAV than in the HC (*p <*0.0001) (**Figure 2A**). The RCN of mTOR in isolated Tregs was not significantly different between the patients with AAV and the HC (*p* = 0.828) (**Figure 2B**), whereas expression of pho-mTOR in Tregs was significantly higher in the patients with AAV than that in the HC (*p* = 0.003) (**Figure 2C**). SIRT1 expression was significantly lower in the patients with AAV than in the HC (*p <*0.0001) (**Figure 2D**).

### Changes in the Intracellular Environment in Tregs After Treatment With RVL

**Figure 3:**
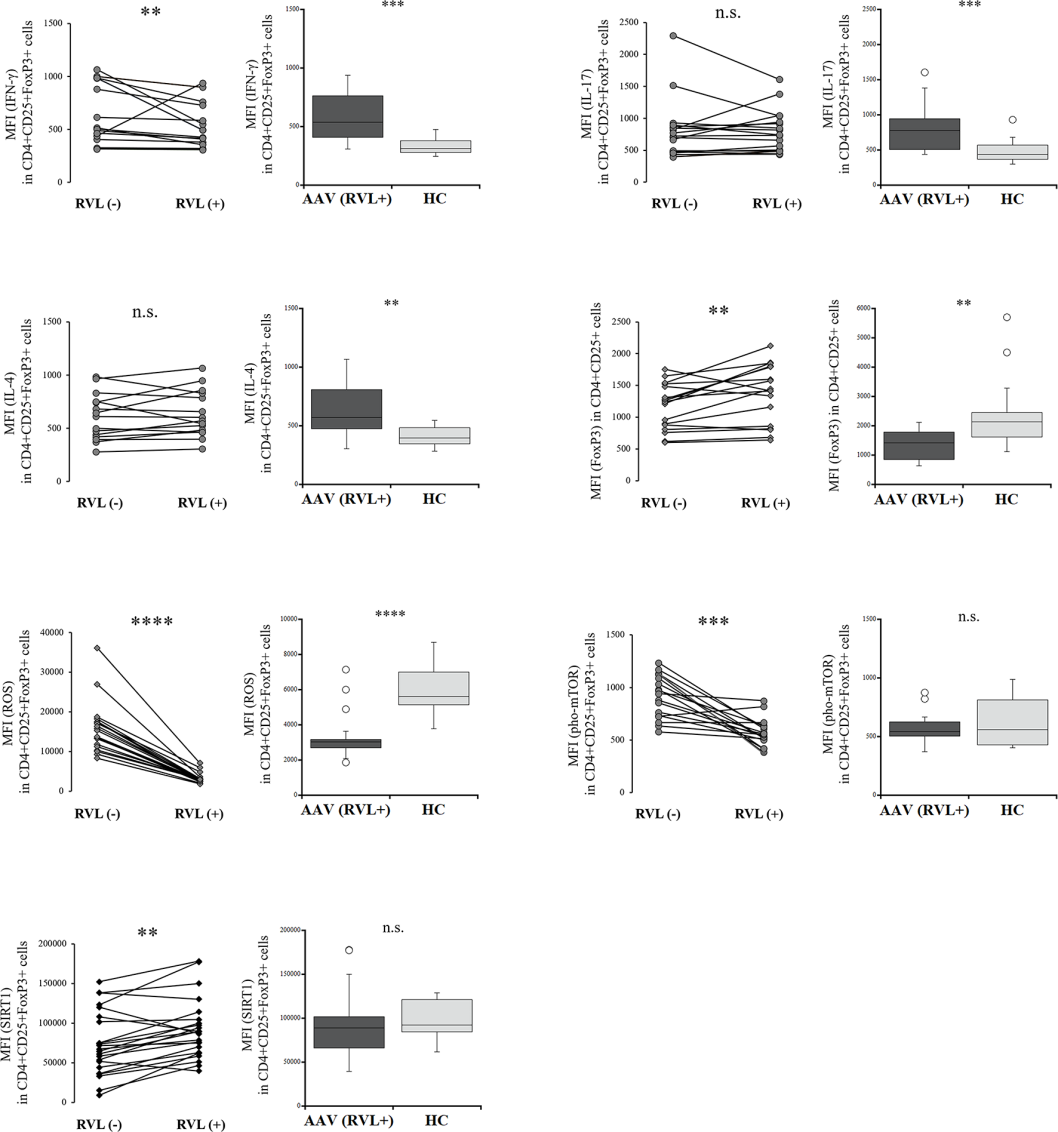
Comparisons of regulatory T cells with and without resveratrol (RVL) treatment in the patients with AAV. Comparisons with and without RVL treatment (left) and those between after RVL treatment in the patients with AAV and healthy controls (HC) (right), with regards to median fluorescence index (MFI) of interferon-γ (IFN-γ), interleukin (IL)-17, or IL-4 in CD4+CD25+FoxP3+ cells (n = 18), MFI of FoxP3 in CD4+CD25+FoxP3+ cells (n = 18), MFI of ROS in CD4+CD25+FoxP3+ cells (n = 20), MFI of phosphorylated mammalian target of rapamycin (pho-mTOR) in CD4+CD25+FoxP3+ cells (n = 18), and MFI of sirtuin 1 (SIRT1) in CD4+CD25+FoxP3+ cells (n = 23). n.s., not significant; **p < 0.005; ***p < 0.001; ****p < 0.0001.

We evaluated the intracellular expression of etiologic factors described above in Tregs with and without RVL treatment in the patients with AAV. IFN-γ expression was significantly decreased in Tregs after RVL treatment (*p* = 0.003) (**Figure 3**), but was significantly higher than in the HC (*p* = 0.0001). When comparing IL-17 and IL-4 expression in Tregs, there was no significant difference with or without RVL treatment. The percent frequencies of IFN-γ, IL-17, and IL-4 positive CD4+CD25+FoxP3+ cells with and without RVL treatment were not significantly different in the patients with AAV (*p* = 0.132, *p* = 0.214, *p* = 0.325, respectively) (**Supplementary Figure 3**). The expression of FoxP3 in CD4+CD25+ cells was significantly increased after RVL treatment (*p* = 0.002), but was lower than in the HC (*p* = 0.002). The production of ROS in Tregs was significantly decreased after RVL treatment (*p* < 0.0001), and was significantly lower than that in the HC (*p* < 0.0001). Additionally, pho-mTOR expression in Tregs was also significantly decreased after RVL treatment (*p* = 0.0006), but the levels were not significantly different from those in the HC (*p* = 0.525). The expression of SIRT1 in Tregs was significantly increased after RVL treatment (*p* = 0.002). In the HC, decreased expression of ROS and pho-mTOR and increased expression of SIRT1 were significantly shown in CD4+CD25+FoxP3+ cells with RVL treatment (*p* = 0.001, *p* = 0.002, *p* = 0.008, respectively), whereas intracellular expression of IFN-γ, IL-17, IL-4, and FoxP3 was not significantly different (**Supplementary Figure 4** **)**.

Hence, RVL could contribute to decreasing ROS expression and phosphorylation of mTOR, as well as increase in SIRT1, in Tregs from the patients with AAV and HC. In addition, decreased expression of IFN-γ in Tregs and their increased expression of FoxP3 after RVL treatment were also demonstrated in the patients with AAV.

### Comparison of the Intracellular Environment in Tregs With and Without SRT1720

**Figure 4:**
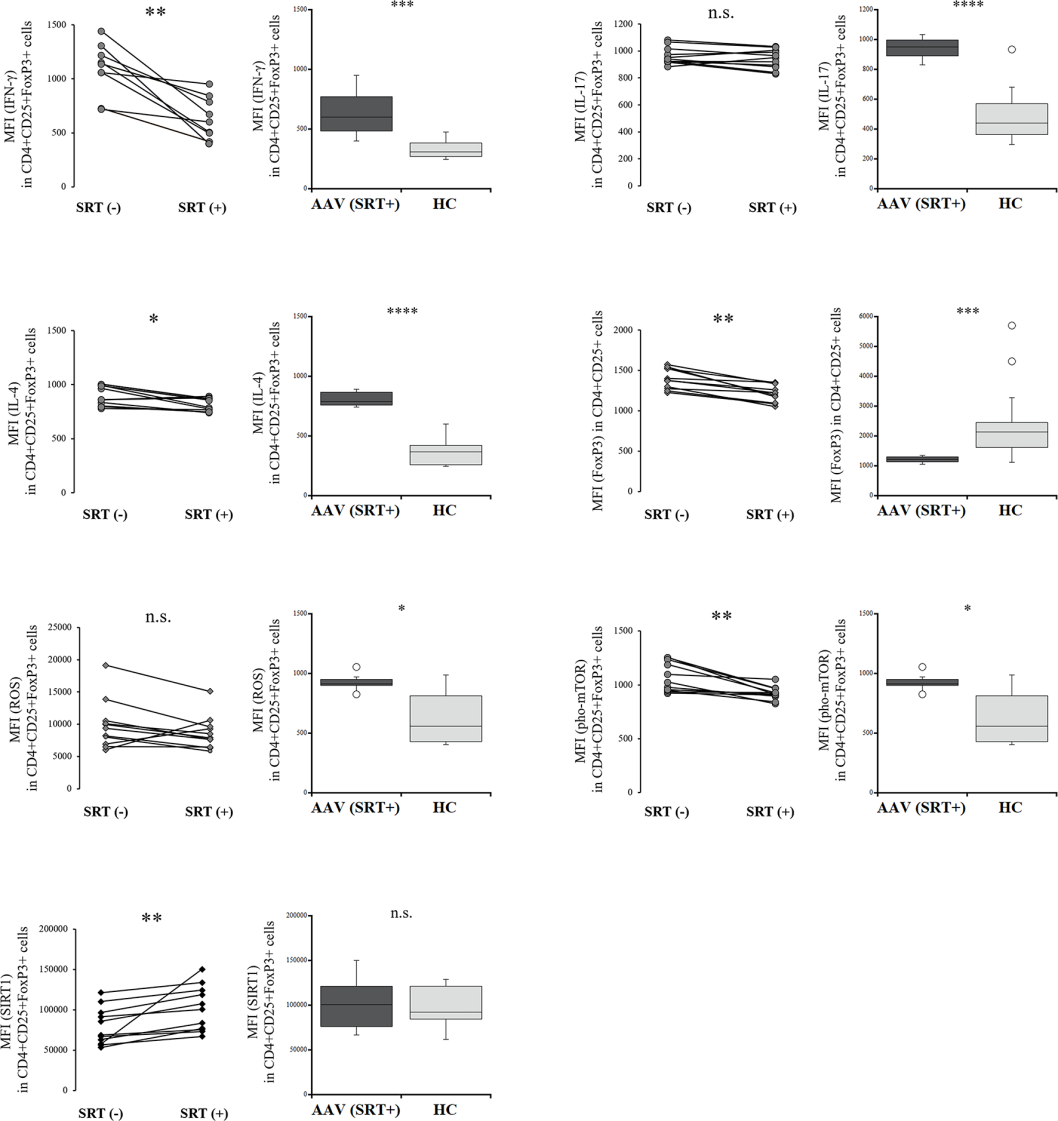
Comparisons of regulatory T cells with and without SRT1720 treatment in the patients with AAV. Comparisons with and without SRT1720 treatment (left) and those between after SRT1720 treatment in the patients with AAV and healthy controls (HC) (right), with regards to median fluorescence index (MFI) of interferon-γ (IFN-γ), interleukin (IL)-17, or IL-4 in CD4+CD25+FoxP3+ cells (n = 11), MFI of FoxP3 in CD4+CD25+FoxP3+ cells (n = 11), MFI of ROS in CD4+CD25+FoxP3+ cells (n = 11), MFI of phosphorylated mammalian target of rapamycin (pho-mTOR) in CD4+CD25+FoxP3+ cells (n = 11), and MFI of sirtuin 1 (SIRT1) in CD4+CD25+FoxP3+ cells (n = 11). n.s., not significant; *p < 0.05; **p < 0.005; ***p < 0.001; ****p < 0.0001.

To determine the differences in Tregs treated with RVL from those in which SIRT1 was selectively activated, we evaluated the intracellular expression of etiologic factors in Tregs with and without SRT1720 treatment. The expression of IFN-γ and IL-4 was significantly decreased in Tregs after SRT1720 treatment (*p* = 0.003 and *p* = 0.013, respectively) (**Figure 4**), but both IFN-γ and IL-4 levels were not significantly different in Tregs treated with SRT1720 compared to those treated with RVL (*p* = 0.393 and *p* = 0.068, respectively). IL-17 levels were not significantly different between Tregs with and without SRT1720 treatment (*p* = 0.182). The percent frequency of IFN-γ+CD4+CD25+FoxP3+ cells was significantly decreased after SRT1720 treatment in patients with AAV (*p* = 0.026), whereas those of IL-17 and IL-4 positive CD4+CD25+FoxP3+ cells with and without SRT1720 treatment were not significantly different in the patients with AAV (*p* = 0.423, *p* = 0.200) (**Supplementary Figure 3**). Meanwhile, the expression of FoxP3 was significantly lower in CD4+CD25+ cells with SRT1720 than that in the cells without SRT1720 treatment (*p* = 0.003). ROS expression in Tregs was not significantly different with or without SRT1720 treatment (*p* = 0.182). The expression of pho-mTOR was significantly decreased in Tregs after SRT1720 treatment (*p* = 0.003), but expression levels of pho-mTOR were significantly higher in Tregs treated with SRT1720 than those in Tregs treated with RVL (*p* < 0.0001). In the HC, decreased expression of IL-4 and pho-mTOR and increased expression of SIRT1 were significantly demonstrated in CD4+CD25+FoxP3+ cells with SRT1720 treatment (*p* = 0.013, *p* = 0.005, *p* = 0.005, respectively), no significant differences in intracellular expression of IFN-γ, IL-17, ROS, and FoxP3 (**Supplementary Figure 4** **)**.

Consequently, SRT1720 could contribute to decreasing phosphorylation of mTOR and increasing IL-4 and SIRT1 in Tregs from the patients with AAV and HC. In the patients with AAV, decreased expression of IFN-γ was also demonstrated in Tregs with SRT1720 treatment. However, Tregs with SRT1720 treatment significantly demonstrated decreased expression of FoxP3; meanwhile, they had no efficacy in reducing ROS.

### Intracellular Expression of IFN-γ, IL-17, and IL-4 in CD4+CD25- Cells and Their Changes With and Without RVL or SRT1720 Treatment

The expression of IFN-γ, IL-17, and IL-4 in CD4+CD25- cells was significantly higher in the patients with AAV than in the HC (*p* < 0.0001, *p* = 0.0019, *p* = 0.0009, respectively) (**Supplementary Figure 5**). In the patients with AAV, IFN-γ expression was significantly decreased in CD4+CD25- cells after RVL and SRT1720 treatment (*p* = 0.016, *p* = 0.041). However, IFN-γ expression in CD4+CD25- cells after RVL and SRT1720 treatment were significantly higher than that in the HC (*p* = 0.0006, *p* = 0.0002).

### Suppression Ability of Tregs With and Without RVL Treatment

**Figure 5:**
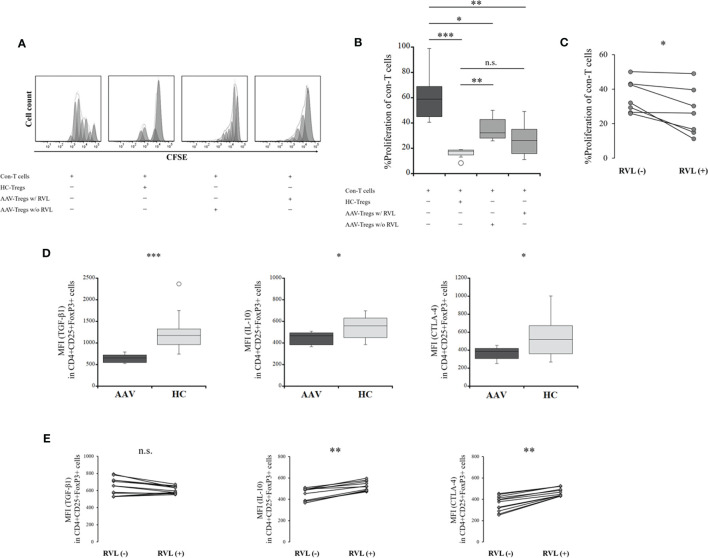
Suppressive assay of regulatory T cells (Tregs) and their co-inhibitory factors before and after treatment with resveratrol (RVL). **(A)** representative histograms showing proliferation of conventional T (Con-T) cells in the absence of Tregs, in the presence of Tregs from the HC (HC-Tregs), in the presence of Tregs from the patients with AAV (AAV-Tregs) with (w/) RVL treatment, and in the presence of AAV-Tregs without (w/o) RVL treatment. **(B)** comparisons of the percent (%) proliferation of Con-T cells in the absence of Tregs (n = 12), in the presence of HC-Tregs (n = 9), AAV-Tregs with RVL (n = 7), and AAV-Tregs without RVL treatment (n = 7). **(C)** alteration of the percent (%) proliferation of Con-T cells with AAV-Tregs before and after RVL treatment (n = 7). **(D)** comparisons of median fluorescence index (MFI) of transforming growth factor (TGF)-β1, IL-10, or cytotoxic T-lymphocyte-associated protein 4 (CTLA-4) in CD4+CD25+FoxP3+ cells between patients with AAV (n = 12) and HC (n = 10). **(E)** alteration of MFI of TGF-β1, IL-10, or CTLA-4 in Tregs from patients with AAV before and after RVL treatment (n = 12). AAV, ANCA-associated vasculitis; HC, healthy controls; n.s., not significant; *p < 0.05; **p < 0.005; ***p < 0.0005.

The proliferation of con-T target cells was evaluated to determine the suppressive ability of Tregs. The proliferation of con-T cells in the presence of Tregs from the HC was significantly lower than that in the absence of Tregs (*p* = 0.0001) (**Figures 5A, B** **)**. The proliferation of con-T cells in the presence of Tregs from the patients with AAV was significantly higher than that in the presence of Tregs from the HC (*p* = 0.0047), demonstrating that the suppressive function of Tregs is impaired in AAV, although the proliferation of con-T cells in the presence of Tregs from AAV was significantly lower than that in the absence of Tregs (*p* = 0.016). Comparing the suppressive ability of Tregs with and without RVL treatment, the proliferation of con-T cells in the presence of Tregs treated with RVL was significantly lower than that in Tregs without RVL (*p* = 0.017) (**Figure 5C**). The proliferation of con-T cells in the presence of Tregs treated with RVL was higher, although not significantly different from that in the presence of Tregs from the HC (*p* = 0.612) (**Figure 5B**).

The expression of co-inhibitory factors of Tregs, including TGF-β1, IL-10, and CTLA-4, was significantly lower in the patients with AAV than in the HC (*p* = 0.0002, *p* = 0.041, *p* = 0.025, respectively) (**Figure 5D**). After treatment with RVL, the expression of IL-10 and CTLA-4 was significantly increased (*p* = 0.002 and *p* = 0.002, respectively), whereas that of TGF-β1 was not significantly different (*p* = 0.059) (**Figure 5E**).

Taken together, the suppressive ability of Tregs and their expression of co-inhibitory factors were significantly lower in the patients with AAV than in the HC. RVL treatment could significantly increase the suppressive ability of Tregs and their expression of IL-10 and CTLA-4. However, no significant finding of TGF-β1 expression was demonstrated in Tregs after RVL treatment.

## Discussion

This study demonstrated a decrease in Tregs together with impaired functional activity in the acute phase of AAV. Some investigations inconsistently indicated decreased, increased, or equivalent expression of Tregs in autoimmune diseases, including AAV, compared to the healthy individuals, whereas dysfunction of Tregs was consistently described (3–5, 7, 21). However, we confirmed imbalanced homeostatic changes in Tregs, in which overexpression of effector cytokines and decreased FoxP3 expression were observed. Furthermore, intracellular increases in oxidative stress and mTOR activation were also demonstrated, suggesting an underlying mechanism for the plasticity and instability of Tregs in AAV. We also assessed how a reduction in SIRT1 is associated with the alterations in Tregs.

The expression of effector cytokines, including IFN-γ, IL-17, and IL-4, was significantly higher in Tregs from the patients with AAV, resulting in plastic changes in Tregs, leading to impaired immune tolerance in the acute phase of AAV. The plasticity of Tregs, which represents effector cytokine expression in the phenotypical population of T cells expressing FoxP3 (6, 7, 22, 23), could be implicated in insufficient suppressive function (6). The expression of FoxP3 in CD4+CD25+ cells was also decreased in AAV, resulting in Tregs failing in their suppressive ability because FoxP3 expression in the conventional phenotype of Tregs plays a pivotal role in regulating extraordinary immune reactions (24, 25). Additionally, downregulation of FoxP3 could be promoted in response to inflammatory signals (26–28); notably, intracellular induction of effector cytokines, including IFN-γ, IL-17, and IL-4, downregulated FoxP3 expression in Tregs (22, 27, 29). Furthermore, Tregs deficient in FoxP3 obtained effector cell function and lost their suppressive ability (23). We postulated that Tregs in AAV, which were diverted into Th-like Tregs, could ultimately lose FoxP3 expression, resulting in diminished suppressive function. In the suppression assay, we evaluated the functional ability of Tregs isolated using a commercially available magnetic isolation kit. Expression of FoxP3 in isolated Tregs was significantly lower in the patients with AAV than that in the HC. Therefore, the defective Tregs function in AAV could be attributable to decreased FoxP3 expression, which could underlie the conversion to Th-like Tregs. Moreover, the plasticity of Tregs could contribute to insufficient immunological tolerance, leading to disease development.

ROS overproduction in Tregs was also a notable result. Excessive oxidative stress is implicated in the pathogenesis of AAV, wherein activated neutrophils release ROS (2), whereas no evidence of Tregs expressing ROS in the immune system underlying the AAV development has been shown to date. Oxidative stress is diversely implicated in the function of the immunocompetent cells (30). Modest levels of ROS are physiologically necessary for immune cell survival; however, high exposure to ROS could negatively impact immunocompetent cell function and activity (10). It has been suggested that the functional ability of Tregs could be dependent on the concentration of ROS, or oxidative stress has been implicated in the activation of cofactors related to Treg suppressive ability (31, 32). Furthermore, plastic alteration of the intracellular environment could also be evoked, depending on ROS concentration (10, 11). In an investigation of type I diabetes, induction of oxidative stress reduced Tregs despite inverse induction in cytotoxic T cells (33). Decreased expression of FoxP3, increased expression of effector cytokines, and abrogated suppressive function were also demonstrated together with significantly higher levels of ROS in Tregs from the patients with AAV, suggesting ROS expression could promote the disability and instability of Tregs in AAV.

Oxidative stress also induces the activation of the mTOR pathway within immunocompetent cells *via* Rheb enhancement (12, 34). The expression of pho-mTOR was significantly higher in Tregs from the patients with AAV. mTOR signaling plays a crucial role in regulating the activity of immunocompetent cells, such as protein synthesis, metabolism, proliferation, growth, and survival as a serine/threonine protein kinase. mTOR signaling activation has also been implicated in inducing the transcription of effector cytokines (12, 35). Moreover, persistent activation of mTOR signaling robustly attenuates the function of Tregs (36). Therefore, mTOR may be a key mediator in determining Treg ability and plasticity in AAV. Conversely, inhibition of the mTOR pathway is required for retaining functional ability and FoxP3 expression of Tregs, while preventing the generation of effector T cells (37, 38). Besides, SIRT1 was significantly decreased in Tregs in AAV, suggesting that SIRT1 deficiency could elicit the instability of Tregs. SIRT1 is also necessary for retaining circulating T cell tolerance (39), and SIRT1 acts as an immune-aging modulator (17). Our results verified a significant reduction in ROS and pho-mTOR levels and increased SIRT1 expression in Tregs after RVL treatment. Additionally, increases in FoxP3 expression and the suppressive function of Tregs were also demonstrated. Herein, we needed to investigate whether selective activation of SIRT1 could also provide same efficacies for retaining homeostatic environment in Tregs. However, selective activation of SIRT1 by treatment with SRT1720, known as the direct activator of SIRT1 (14), ultimately resulted in reduced FoxP3 expression in our study. Previous studies have demonstrated the downregulation of FoxP3 by directly activating SIRT1 (40, 41), supporting our experimental results. Moreover, Tregs treated with SRT1720 showed significant overproduction of ROS and lesser reduction in pho-mTOR levels than those treated with RVL. The current results demonstrated that efficient redox homeostasis provides more potent suppression of mTOR phosphorylation than direct SIRT1 activation. RVL indirectly affects the activation of SIRT1; namely, RVL stimulates AMPK, which increases SIRT1 activity by activating the upstream kinase of AMPK, known as LKB1 (14, 42, 43). SIRT1 could also activate AMPK by interacting with LKB1, even in the absence of RVL (43). AMPK is a negative regulator of mTOR (13), suggesting that the regulation of mTOR by RVL treatment or SIRT1 activation could be mediated through AMPK activation. Metformin, which is used to treat diabetes mellitus, also plays a role in negatively regulating mTOR activation by stimulating AMPK (44). NAC (*N*-acetylcysteine), also known as an antioxidant, improved disease activity by suppressing mTOR activation in SLE (45, 46). Moreover, it explained that NAC suppresses induction of ROS, indirectly resulting in a reduction in mTOR activation. Accordingly, RVL may provide integrated roles of negatively regulating ROS-mediated and AMPK signaling, such as a combination of NAC and metformin, in repressing mTOR activation within Tregs from the patients with AAV. Taken together, the instability of Tregs in AAV could be promoted by induced phosphorylation of mTOR, in which intracellular overproduction of ROS could be more prominently implicated than reduced SIRT1 activity.

There were some limitations to this study. RVL may possess the antioxidant ability and suppress mTOR activation, leading to increased FoxP3 expression and functional ability in Tregs. However, the stability of Tregs after RVL treatment in patients was less than that in HC. Plastic changes in Tregs exhibiting increases in IFN-γ, IL-17, and IL-4 expression remained, suggesting that a fundamental immunosuppressive therapy could be required to suppress the inflammatory signal promoting the Th-like shift. Hence, RVL could be useful as adjuvant therapy in partial remission after administering immunosuppressive drugs. Deficient expression of TGF-β1, IL-10, and CTLA-4, which are known as potential mediators that facilitate the suppressive ability of Tregs (21), was also observed in Tregs from the patients with AAV. The expression of these co-inhibitory mediators of Tregs could be negatively regulated by activating mTOR signaling (47). Besides, TGF-β1 promotes the downregulation of mTOR signaling and contributes to maintaining the stability of Tregs and FoxP3 expression (48). Intracellular production of TGF-β1 and CTLA-4 is implicated in retaining Treg ability, whereas the process of TGF-β1 production differed from that of CTLA-4 (47, 49). Tregs ultimately showed a significant increase in the expression of IL-10 and CTLA-4 after RVL treatment, despite not being significantly different from that of TGF-β1, suggesting that the efficacy of RVL has a limitation especially for treating TGF-β1 expression in Tregs. Therefore, further studies are required to investigate the mechanism underlying the stabilization of Tregs. Besides, this study only evaluated efficacies of RVL *in vitro*. Therefore, it is also necessary to perform the clinical research, in which RVL will be administered to the patients with AAV, to develop this attempt for practical application.

In conclusion, Tregs from the patients with AAV showed increased IFN-γ, IL-17, and IL-4 expression, decreased FoxP3 expression, and impaired functional activity. Imbalanced changes in Tregs could be attributed to induced phosphorylation of mTOR, which is predominantly facilitated by intracellular overproduction of ROS. Reduced SIRT1 activity was also observed in Tregs from the patients with AAV, but direct activation of SIRT1 ultimately resulted in reduced FoxP3 expression. Additionally, RVL was significantly effective in promoting a reduction in ROS expression with dephosphorylation of mTOR in Tregs, thereby contributing to the increased FoxP3 expression and functional activity. This study suggests that ROS play a pivotal role in inducing plasticity and impaired functional activity of Tregs in AAV. RVL could be useful as an assisting therapy unless conventional immunosuppressive treatment is sufficient to restore Treg stability. However, their imbalanced homeostatic changes, including higher effector cytokines, lower FoxP3 expression and functional activity than in the HC, remained even after RVL treatment, suggesting that more divergent mechanisms are involved in the imbalance of Treg homeostasis. It is necessary to investigate more precise signaling mechanisms underlying the instability of Tregs in AAV.

## Data Availability Statement

The raw data supporting the conclusions of this article will be made available by the authors, without undue reservation.

## Ethics Statement

The studies involving human participants were reviewed and approved by the Ethics Committee of Shinshu University (approval number: 614). The patients/participants provided their written informed consent to participate in this study.

## Author Contributions

All authors made the design of this study, developed the structure and argument for this study. YSh, DK, TI, RT, and SN recruited blood samples and clinical data. YSh performed laboratory investigations, and analyzed obtained data. YSh prepared the draft of this manuscript. YSh and YSe contributed to revise the manuscript. All authors contributed to the article and approved the submitted version.

## Funding

This study was supported by JSPS KAKENHI Grant Number JP18K08385 and The Association for Fordays Self-Reliance Support in Japan. The funders were not involved in the study design, collection, analysis, interpretation of data, the writing of this article or the decision to submit it for publication.

## Conflict of Interest

The authors declare that the research was conducted in the absence of any commercial or financial relationships that could be construed as a potential conflict of interest.

## Publisher’s Note

All claims expressed in this article are solely those of the authors and do not necessarily represent those of their affiliated organizations, or those of the publisher, the editors and the reviewers. Any product that may be evaluated in this article, or claim that may be made by its manufacturer, is not guaranteed or endorsed by the publisher.
