# Supplementary material for: Oxidative Stress Promotes Instability of Regulatory T Cells in Antineutrophil Cytoplasmic Antibody-Associated Vasculitis
Source: Front Immunol. 2021 Dec 7;12:789740. doi: 10.3389/fimmu.2021.789740 (PMC8691772; doi:10.3389/fimmu.2021.789740)
Supplement: Supplementary file 1 [file DataSheet_1.pdf]

Supplementary figure 1

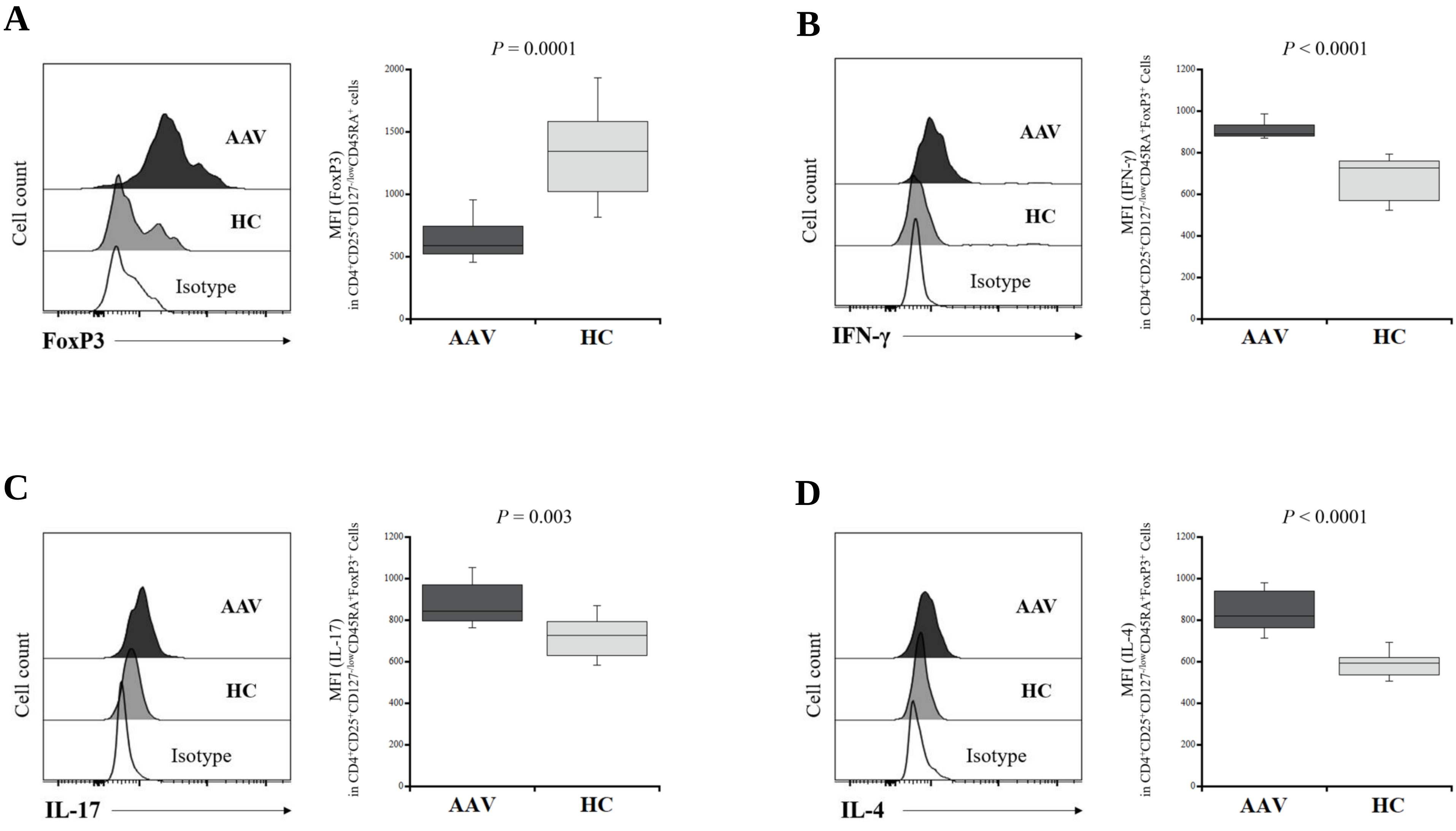

Comparisons of FoxP3 expression in $CD4^{+}CD25^{+}CD127^{-/low}CD45RA^{+}$ cells and their intracellular effector cytokine expression between the patients with AAV and healthy controls (HC).

(A) The median fluorescence index (MFI) of FoxP3 in $CD4^{+}CD25^{+}CD127^{-/low}CD45RA^{+}$ cells. **(B**, **C**, **D)** MFI of effector cytokines, including interferon (IFN)-γ, interleukin (IL)-17, and IL-4, respectively, in $CD4^{+}CD25^{+}CD127^{-/low}CD45RA^{+}FoxP3^{+}$ cells. The Mann-Whitney U test was used for comparisons between the patients with AAV (n = 12) and HC (n = 10).

Supplementary figure 2

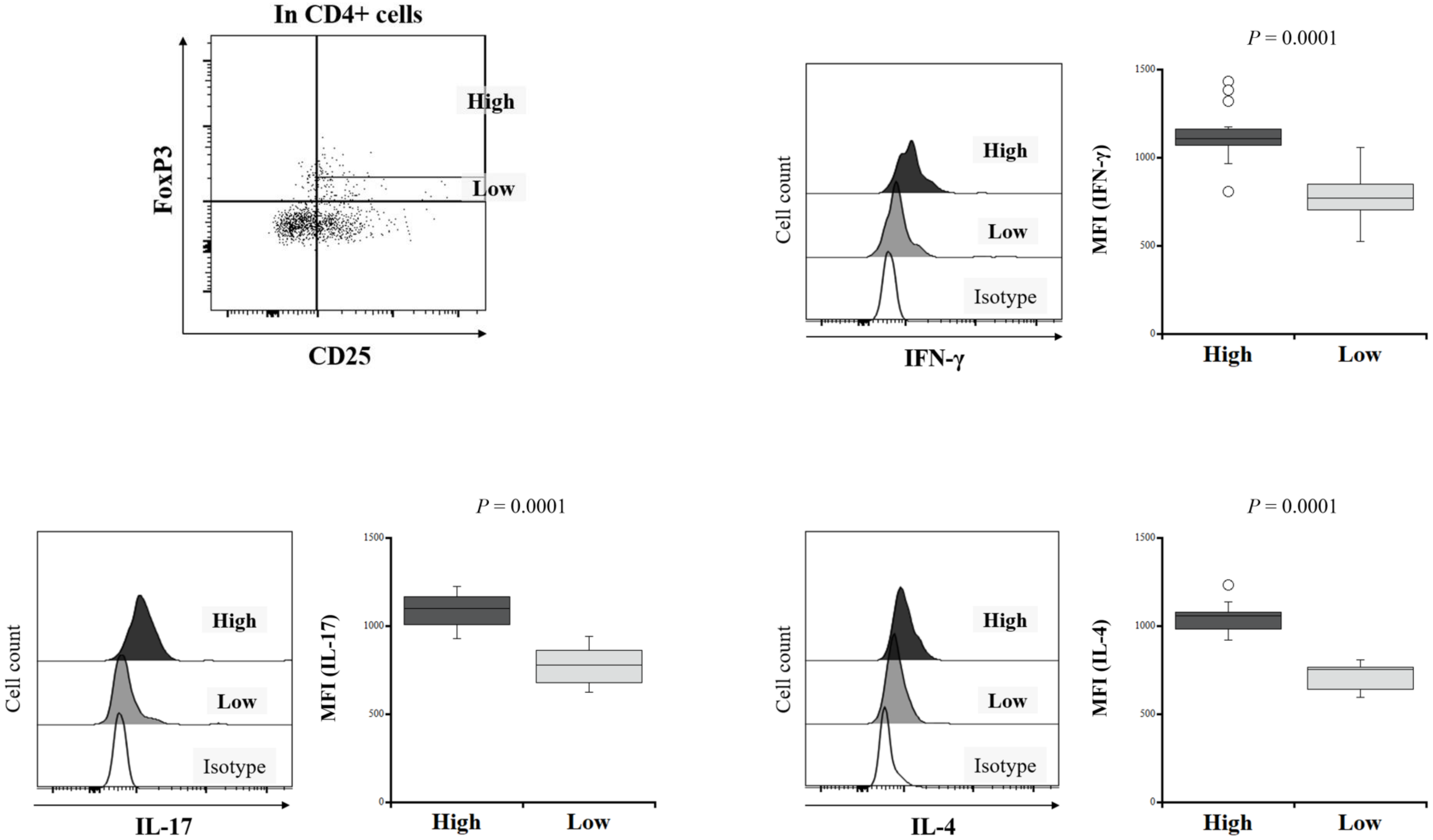

Intracellular expression of IFN-γ, IL-17, and IL-4 in high-and low-density expression of FoxP3 in the patients with AAV.

The median fluorescence index (MFI) of IFN-γ, IL-17, and IL-4 were compared in $CD4^+CD25^+FoxP3^{high}$ and $CD4^+CD25^+FoxP3^{low}$ population. High, $CD4^+CD25^+FoxP3^{high}$ cells; Low, $CD4^+CD25^+FoxP3^{low}$ cells. The Wilcoxon's signed-rank test was used for comparisons in patients with AAV (n = 18).

Supplementary figure 3

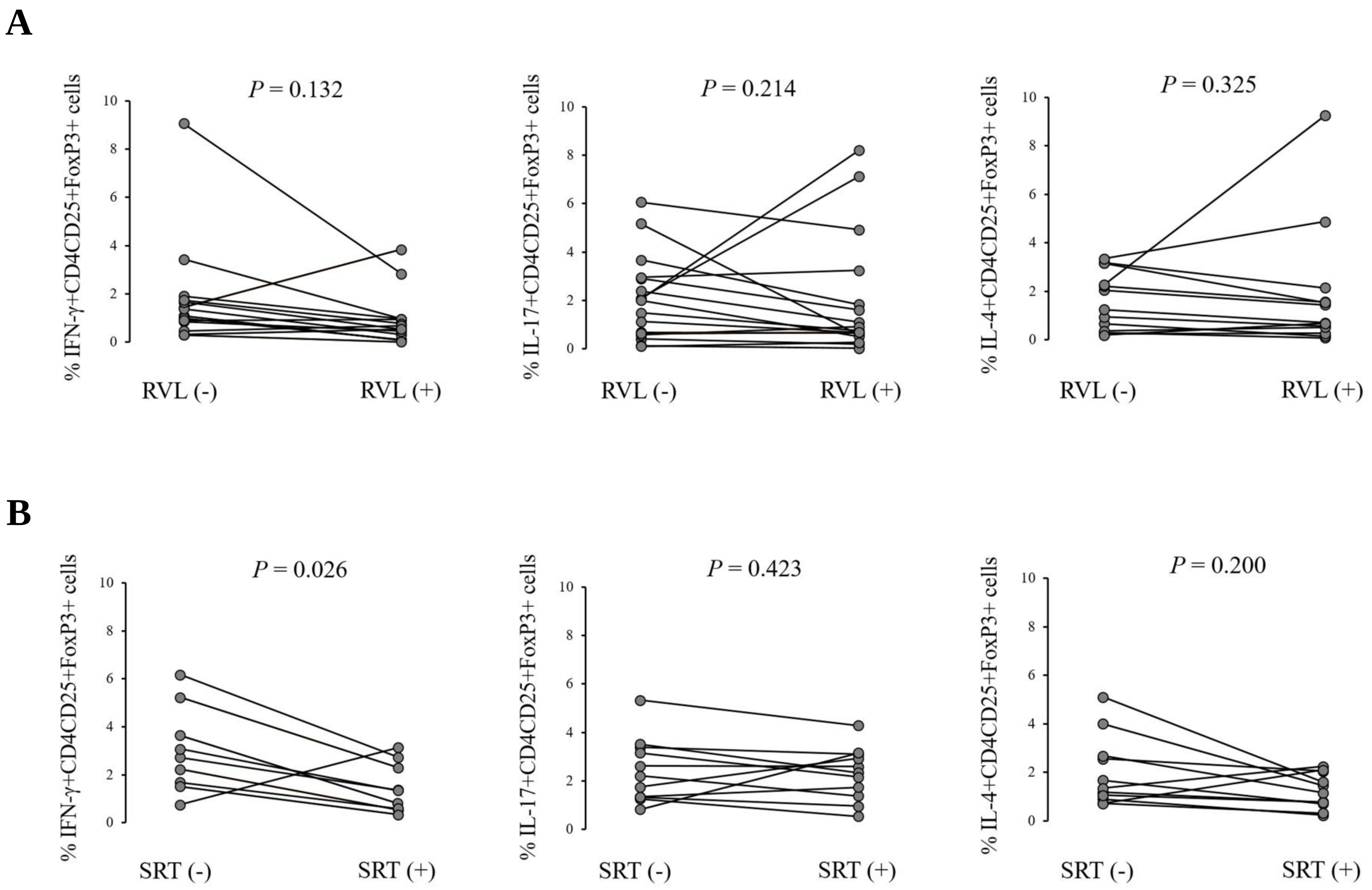

Alterations of IFN-γ, IL-17, and IL-4 expressing $CD4^+CD25^+FoxP3^+$ cells with and without resveratrol or SRT1720 treatment in the patients with AAV.

(**A**) Percent frequencies of IFN-γ, IL-17, and IL-4 positive $CD4^+CD25^+FoxP3^+$ cells with and without resveratrol (RVL) treatment were compared in the patients with AAV (n = 18). (**B**) Percent frequencies of IFN-γ, IL-17, and IL-4 positive $CD4^+CD25^+FoxP3^+$ cells with and without SRT1720 (SRT) treatment were compared in the patients with AAV (n = 11). The Wilcoxon's signed-rank test was used for comparisons.

Supplementary figure 4

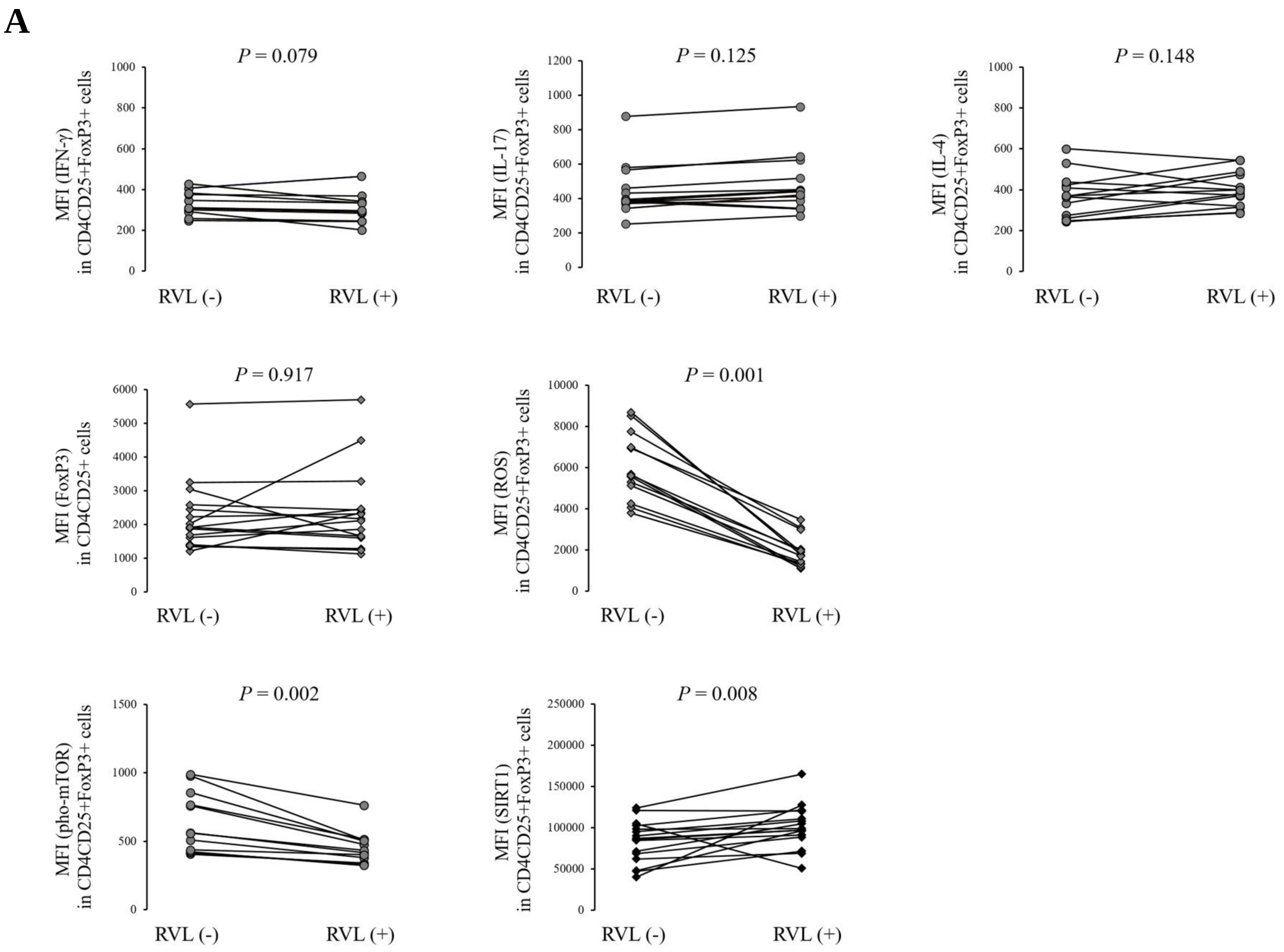

B

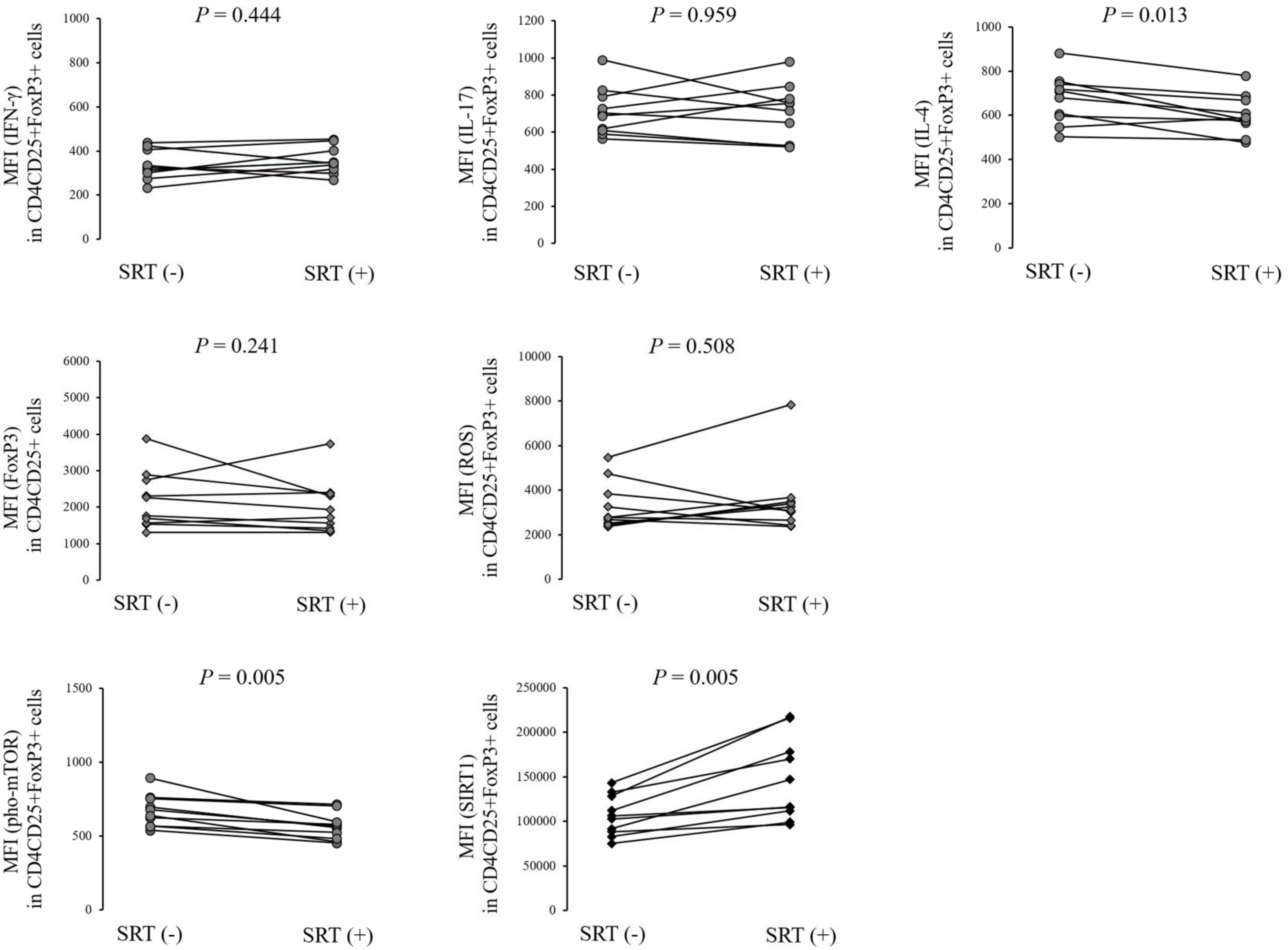
P = 0.444
MFI (IFN-γ) in CD4CD25+FoxP3+ cells
1000
800
600
400
200
0
SRT (-)
SRT (+)
P = 0.959
MFI (IL-17) in CD4CD25+FoxP3+ cells
1200
1000
800
600
400
200
0
SRT (-)
SRT (+)
P = 0.013
MFI (IL-4) in CD4CD25+FoxP3+ cells
1000
800
600
400
200
0
SRT (-)
SRT (+)
P = 0.241
MFI (FoxP3) in CD4CD25+ cells
6000
5000
4000
3000
2000
1000
0
SRT (-)
SRT (+)
P = 0.508
MFI (ROS) in CD4CD25+FoxP3+ cells
10000
8000
6000
4000
2000
0
SRT (-)
SRT (+)
P = 0.005
MFI (pho-mTOR) in CD4CD25+FoxP3+ cells
1500
1000
500
0
SRT (-)
SRT (+)
P = 0.005
MFI (SIRT1) in CD4CD25+FoxP3+ cells
250000
200000
150000
100000
50000
0
SRT (-)
SRT (+)

Alterations of intracellular environment in Tregs with and without resveratrol or SRT1720 treatment in the healthy controls.

(**A**) The median fluorescence index (MFI) of IFN-γ, IL-17, IL-4, reactive oxygen species (ROS), phosphorylated mTOR (pho-mTOR), and sirtuin 1 (SIRT1) in $CD4^+CD25^+FoxP3^+$ cells with and without resveratrol (RVL) treatment were compared in the healthy controls (HC). MFI of FoxP3 in $CD4^+CD25^+$ cells with and without RVL treatment was also compared in the HC. (**B**) MFI of IFN-γ, IL-17, IL-4, ROS, pho-mTOR, and SIRT1 in $CD4^+CD25^+FoxP3^+$ cells with and without SRT1720 (SRT) treatment were compared in the HC. MFI of FoxP3 in $CD4^+CD25^+$ cells with and without SRT treatment was also compared in the HC. The Wilcoxon's signed-rank test was used for comparisons with and without RVL or SRT treatment.

Supplementary figure 5

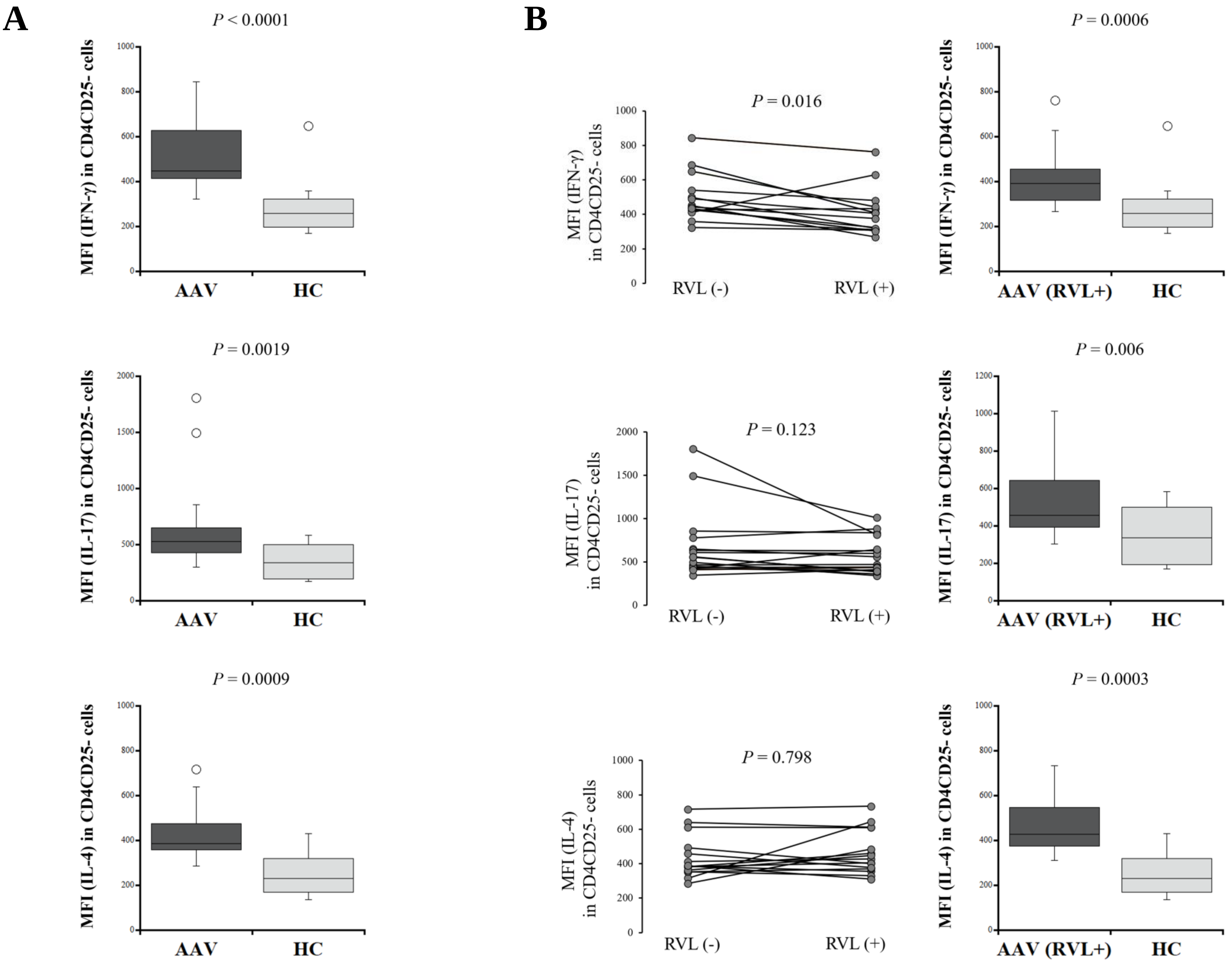

C

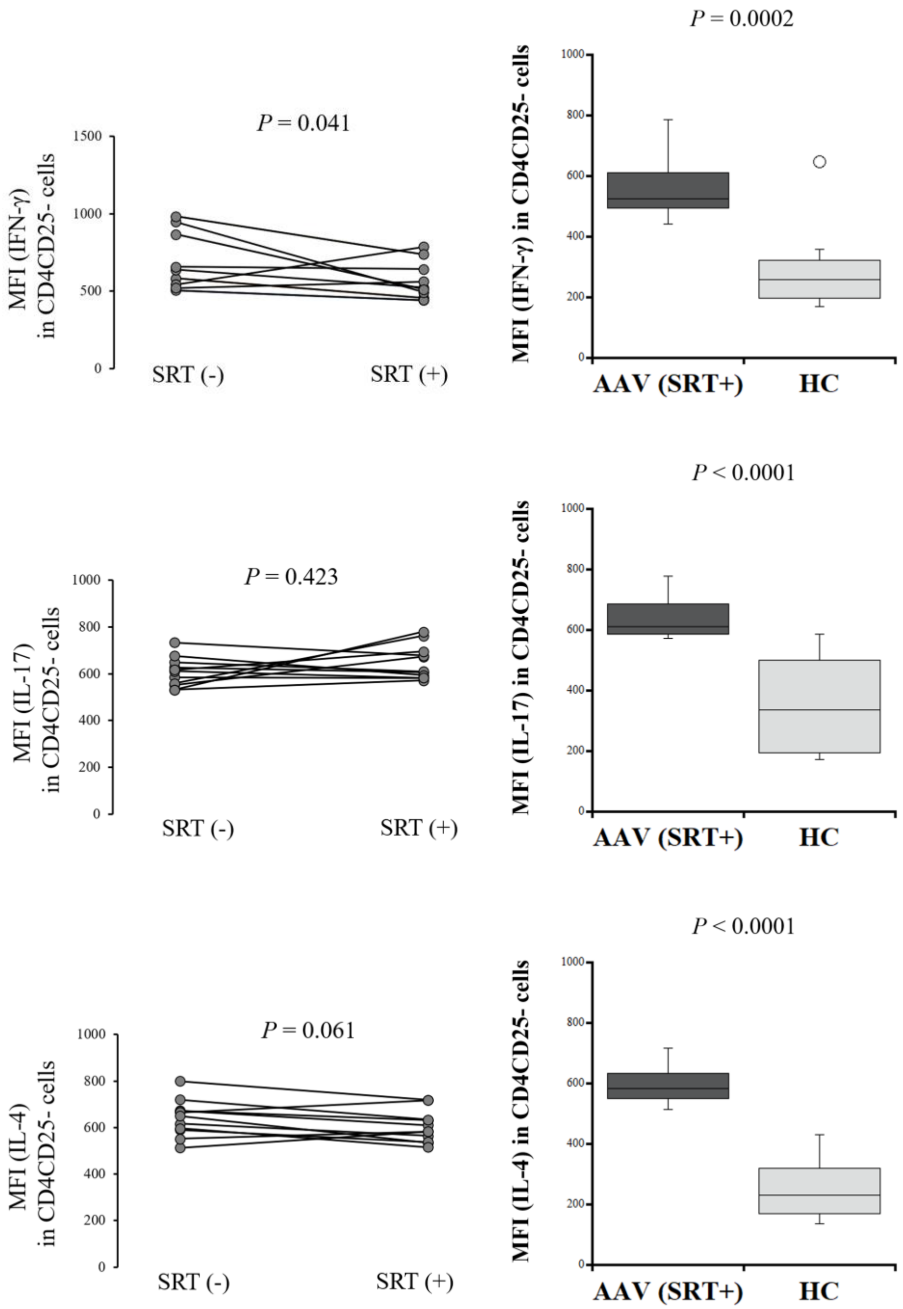

P = 0.041
MFI (IFN-γ) in CD4CD25- cells
SRT (-)
SRT (+)
P = 0.0002
MFI (IFN-γ) in CD4CD25- cells
AAV (SRT+)
HC
P = 0.423
MFI (IL-17) in CD4CD25- cells
SRT (-)
SRT (+)
P < 0.0001
MFI (IL-17) in CD4CD25- cells
AAV (SRT+)
HC
P = 0.061
MFI (IL-4) in CD4CD25- cells
SRT (-)
SRT (+)
P < 0.0001
MFI (IL-4) in CD4CD25- cells
AAV (SRT+)
HC

Intracellular expression of IFN-γ, IL-17, and IL-4 in $CD4^+CD25^-$ cells with and without resveratrol or SRT1720 treatment in the patients with AAV.

(**A**) The median fluorescence index (MFI) of IFN-γ, IL-17, and IL-4 in $CD4^+CD25^-$ cells were compared between the patients with AAV (n = 18) and healthy controls (HC) (n = 15). (**B**) Alterations of MFI of IFN-γ, IL-17, and IL-4 in $CD4^+CD25^-$ cells before and after treatment with resveratrol (RVL) were shown in the patients with AAV (n = 18) (left). MFI of IFN-γ, IL-17, and IL-4 in $CD4^+CD25^-$ cells with RVL treatment in the patients with AAV were compared to those in the HC (right). (**C**) Alterations of MFI of IFN-γ, IL-17, and IL-4 in $CD4^+CD25^-$ cells before and after treatment with SRT1720 (SRT) were shown in patients with AAV (n = 11) (left). MFI of IFN-γ, IL-17, and IL-4 in $CD4^+CD25^-$ cells with SRT treatment in the patients with AAV were compared to those in the HC (right). The Mann-Whitney U test was used for comparisons between the patients with AAV and HC. The Wilcoxon's signed-rank test was used for comparisons with and without RVL or SRT treatment.
