# Supplementary material for: Oxidative Stress Promotes Instability of Regulatory T Cells in Antineutrophil Cytoplasmic Antibody-Associated Vasculitis
Source: Front Immunol. 2021 Dec 7;12:789740. doi: 10.3389/fimmu.2021.789740 (PMC8691772; doi:10.3389/fimmu.2021.789740)
Supplement: Supplementary file 2 [file Table_1.docx]

**Supplementary Table 1** Information of materials

|  | **Materials** | **Catalog number** | **Vendor** |
| --- | --- | --- | --- |
| Cell isolation Kit | |  |  |
|  | CD4+CD25+ regulatory T cell isolation kit | 130-091-301 | Miltenyi Biotec |
| Antibodies for flow cytometry | |  |  |
|  | PE/Cy7-conjugated anti-CD4 | 300521 | BioLegend |
|  | PC5-conjugated anti-CD25 | IM2646 | Beckman Coulter |
|  | PE-conjugated anti-CD152 | IM2282 | Beckman Coulter |
|  | FITC-conjugated anti-IFN-γ | IM2716U | Beckman Coulter |
|  | PE-conjugated anti-IL-17 | 560486 | BD Biosciences |
|  | PE-conjugated anti-IL-4 | IM2719U | Beckman Coulter |
|  | FITC-conjugated anti-TGF-β1 | 349605 | BioLegend |
|  | PE-conjugated anti-IL-10 | 501403 | BioLegend |
|  | PE-conjugated mTOR (pS2448) | 563489 | BD Biosciences |
|  | FITC-conjugated anti-FoxP3 | 320106 | BioLegend |
|  | Pacific blue-conjugated anti-FoxP3 | 320116 | BioLegend |
|  | PE-conjugated anti-FoxP3 | 560046 | BD Biosciences |
|  | Alexa Fluor 405-conjugated anti-SIRT1 | NBP1-51641AF405 | Novus Biologicals |
|  | CellROX Deep Red Reagent | C10422 | Invitrogen |
|  | CellTrace CFSECell Proliferation Kit | C34554 | Invitrogen |

**Supplementary Table 2** Epidemiologic and clinical findings in patients with AAV

|  |  | **AAV** | **HC** | ***p* value*** |
| --- | --- | --- | --- | --- |
|  | Number | 25 | 17 |  |
|  | Mean age (years) | 63 ± 11 | 58 ± 11 | 0.115 |
|  | Sex (M/F) | 8/17 | 7/10 | 0.387 |
| **Classification**, n (%) | |  |  |  |
|  | MPA | 14 (56) |  |  |
|  | GPA | 11 (44) |  |  |
| **Symptoms**, n (%) | |  |  |  |
|  | Fever (more than 38℃) | 14 (56) |  |  |
|  | Arthritis/arthralgia | 5 (20) |  |  |
|  | Myalgia | 1 (4) |  |  |
|  | Weight loss (more than 2 kg) | 7 (28) |  |  |
|  | Cutaneous | 7 (28) |  |  |
|  | Mucous membranes/eyes | 3 (12) |  |  |
|  | ENT | 11 (44) |  |  |
|  | Pulmonary system | 17 (68) |  |  |
|  | Cardiovascular system | 2 (8) |  |  |
|  | Abdominal system | 1 (4) |  |  |
|  | Renal system | 15 (60) |  |  |
|  | Nervous system | 13 (52) |  |  |
| **BVAS** | | 19.2 ± 6.7 |  |  |
| **Laboratory data** | |  |  |  |
|  | MPO-ANCA, n (%) | 20 (80) |  |  |
|  | PR3-ANCA, n (%) | 5 (20) |  |  |
|  | White blood cells, /µL | 11316 ± 4055 |  |  |
|  | Lymphocytes, /µL | 1325 ± 837 |  |  |
|  | C-reactive protein, mg/dL | 9.2 ± 5.5 |  |  |
|  | ESR, mm/h | 70 ± 31 |  |  |

AAV, antineutrophil cytoplasmic antibody (ANCA)-associated vasculitis; HC, healthy controls; ENT, ear, nose, and throat; BVAS, Birmingham Vasculitis Activity Score version 3 (1); MPO, myeloperoxidase; PR3, proteinase 3; ESR, erythrocyte sedimentation rate.

Data are presented as mean ± SD. *Comparisons between AAV patients and HC using the Mann-Whitney U test for age and Fisher’s exact probability test for sex.

1. Mukhtyar C, Lee R, Brown D, Carruthers D, Dasgupta B, Dubey S, et al. Modification and validation of the Birmingham Vasculitis Activity Score (version 3). Ann Rheum Dis. 2009;68:1827-32.

**Supplementary Table 3** Frequencies of CD4+CD25+CD127-/lowCD45RA+FoxP3+ cells and their intracellular cytokines expression

|  |  | **AAV** | **HC** | ***p* value*** |
| --- | --- | --- | --- | --- |
|  |  | (n = 12) | (n = 10) |  |
| In total lymphocytes | |  |  |  |
|  | CD4+CD25+CD127-/lowCD45RA+FoxP3+ cells | 4.46 ± 2.21 | 7.54 ± 3.15 | 0.025 |
| In CD4+CD25+CD127-/lowCD45RA+FoxP3+ cells | |  |  |  |
|  | %IFN-γ | 44.15 ± 6.01 | 2.90 ± 4.48 | <0.0001 |
|  | %IL-17 | 43.75 ± 12.77 | 3.92 ± 6.47 | <0.0001 |
|  | %IL-4 | 44.79 ± 16.29 | 1.87 ± 2.63 | <0.0001 |

AAV, antineutrophil cytoplasmic antibody-associated vasculitis; HC, healthy controls; IFN-γ, interferon-γ; IL, interleukin.

Data are presented as mean ± SD. *Comparisons between AAV patients and HC using the Mann-Whitney U test.
